# Cloning and Characterization of a *Weissella confusa* Dextransucrase and Its Application in High Fibre Baking

**DOI:** 10.1371/journal.pone.0116418

**Published:** 2015-01-20

**Authors:** Ilkka Kajala, Qiao Shi, Antti Nyyssölä, Ndegwa Henry Maina, Yaxi Hou, Kati Katina, Maija Tenkanen, Riikka Juvonen

**Affiliations:** 1 VTT Technical Research Centre of Finland, Espoo, Finland; 2 Department of Food and Environmental Sciences, University of Helsinki, Helsinki, Finland; Tsinghua University, CHINA

## Abstract

Wheat bran offers health benefits as a baking ingredient, but is detrimental to bread textural quality. Dextran production by microbial fermentation improves sourdough bread volume and freshness, but extensive acid production during fermentation may negate this effect. Enzymatic production of dextran in wheat bran was tested to determine if dextran-containing bran could be used in baking without disrupting bread texture. The *Weissella confusa* VTT E-90392 dextransucrase gene was sequenced and His-tagged dextransucrase Wc392-rDSR was produced in *Lactococcus lactis*. Purified enzyme was characterized using ^14^C-sucrose radioisotope and reducing value-based assays, the former yielding *K*_m_ and *V*_max_ values of 14.7 mM and 8.2 μmol/(mg∙min), respectively, at the pH optimum of 5.4. The structure and size of *in vitro* dextran product was similar to dextran produced *in vivo*. Dextran (8.1% dry weight) was produced in wheat bran in 6 h using Wc392-rDSR. Bran with and without dextran was used in wheat baking at 20% supplementation level. Dextran presence improved bread softness and neutralized bran-induced volume loss, clearly demonstrating the potential of using dextransucrases in bran bioprocessing for use in baking.

## Introduction

Microbial exopolysaccharides (EPS) are extracellular polymers that can be divided into hetero- and homopolysaccharides depending on whether the polymer is composed of several different or single types of monomers. Bacterial homopolysaccharides consist either of D-glucopyranosyl or D-fructofuranosyl subunits and are referred to as glucans and fructans, respectively. EPS may aid in cell adhesion and serve a protective function for the bacterial cells. Some lactic acid bacteria (LAB) produce extracellular dextransucrases, also known as glucosyltransferases (EC 2.4.1.5), which catalyse the hydrolysis of sucrose and the polymerisation of the released glucosyl units into dextran [1]. The production of polymeric dextran is the main enzymatic reaction, but the glucosyl moiety can also be transferred to other acceptors such as maltose. When the concentration of other acceptor substrates is low, water may also act as an acceptor, resulting in the hydrolysis of sucrose [1].

Microbial EPS have commercial value as stabilizing and emulsifying agents in different food products as well as in cosmetics and in medicine [2]. Particularly dextrans, the α-(1→6)-linked glucans with α-(1→2)-, α-(1→3)- and/or α-(1→4)-branches, have multiple potential applications in the food industry, where they could be used as thickening, viscosifying and emulsifying agents. Dextrans are also soluble fibres with potential health benefits such as prebiotic activity [1].

Sourdough is a pre-dough fermented with LAB and yeasts. Fermentation results in the formation of acids and other metabolites, which may lengthen the shelf life and improve the flavour and textural qualities of the bread. In wheat and rye sourdough bread, the LAB-produced dextran can increase the bread volume and improve moisture retention resulting in improved freshness of bread during storage [3, 4]. Furthermore, dextran sourdoughs promote increased softness of crumb texture during storage indicating reduced staling [3]. Many LAB strains indigenous to cereals are capable of dextran production. The positive technological effects of dextran on sourdough bread are correlated with high molecular mass and a low degree of branching of the dextran molecule [4]. LAB belonging to genera *Weissella*, and to the species *W. cibaria* and *W*. *confusa* in particular, have been reported to produce dextran with a molar mass of at least 10^6^ g/mol and typically only 2–4% of α-(1→3)-linkages [5–7].

Sourdough technology is widely used in rye bread production, but is less commonly exploited in wheat baking, since acidity negatively affects the taste of wheat bread [8]. Extensive acidification will counteract the positive impact of *in situ* produced dextran on the bread quality [9]. High dextran content and minimal acidification should therefore be produced during fermentation to achieve maximal technological benefits in the final product [3]. Some *W. confusa* strains have been shown to be efficient dextran producers both under laboratory conditions and in sourdough. In addition, these strains produce less organic acids because they lack the ability to convert fructose into mannitol and acetate [3, 10]. *W. confusa* VTT E-90392 has been reported to produce up to 16 g/kg dextran in wheat sourdough [3], whereas with *W. cibaria* strains, dextran production levels of 0.8–5.8 g/kg have been achieved [10–12].

Wheat bran, a by-product of the dry milling of wheat, is an ingredient rich in dietary fibre, vitamins, minerals and phytochemicals such as antioxidants. It has been shown conclusively that wheat bran is beneficial for gastrointestinal health and diets rich in cereal fibre reduce the risk of certain chronic diseases [13]. However, bread baked with amounts of bran that will benefit health is usually smaller in volume and has increased hardness as well as poorer texture when compared to bread without bran. Furthermore, when used in larger quantities, wheat bran imparts unpleasant mouth-feel to the bread [14]. The hydrocolloidal properties of dextran could facilitate a more substantial use of wheat bran and counter the negative effects of bran on bread quality. Potential to produce glucan in bran matrix has previously been demonstrated using a *Lactobacillus reuteri* starter strain [9].

By using dextransucrase as the catalyst, dextran could be produced in high quantities in the bran-containing wheat bread without fermentation and the associated acid formation. Characterization of dextransucrases and their dextran products is an essential step towards better understanding of the *in situ* homopolysaccharide production in food applications. Previously, dextransucrases from three *Weissella* strains have been cloned and characterized [6, 15, 16]. In the present study, the dextransucrase gene from *W. confusa* VTT E-90392 was identified and cloned. The strain performs well in wheat sourdough baking and its dextran structure is well-characterized [3, 7, 17]. In addition to the *in silico* analysis of the native dextransucrase WcE392-DSR, biochemical characterization of the recombinant enzyme (WcE392-rDSR) and its dextran product was carried out. WcE392-rDSR was used to study *in vitro* dextran production in wheat bran matrix. Furthermore, a new and rapid method for improving the quality of high fibre wheat bread was presented by producing dextran enzymatically in wheat bran before using it in wheat baking.

## Materials and Methods

### Bacterial strains and growth media

*W. confusa* VTT E-90392 (VTT Culture Collection, Espoo, Finland) was cultivated anaerobically in MRS medium [18] (Oxoid, Basingstoke, United Kingdom) at 30°C. The anaerobic conditions were created in airtight jars with Anoxomat Mark II AN2CTS system (Mart Microbiology, Lichtenvoorde, the Netherlands) with 85% nitrogen, 10% hydrogen and 5% carbon dioxide atmosphere. To induce dextran production, 20 g/l sucrose was added to the medium. *Lactococcus lactis* NZ9800[19] that was used as a cloning and expression host was cultivated aerobically in M17 medium supplemented with 0.5% (w/v) glucose (gM17) at 30°C. A nisin-inducible expression vector pNZ8037 [NIZO Food Research, Ede, the Netherlands [19]] was used for heterologous expression. Ten µg/mL chloramphenicol and 4 ng/mL nisin were added to gM17 for vector selection and expression induction, respectively.

### Sequencing and cloning of the gene encoding WcE392-DSR

*L. lactis* plasmid DNA was isolated using the QIAprep Spin Miniprep Kit (Qiagen, Hilden, Germany) following the instructions provided by the manufacturer with the exception that the cells were suspended in 250µl of resuspension buffer P1 supplemented with 2 mg/mL lysozyme and 20 U/mL mutanolysin and incubated for 1 h at 37°C prior to the cell lysis.

Genomic DNA, isolated with the FastDNA Spin Kit for Soil with FastPrep-24 instrument (MP Biomedicals, Solon, OH), was used as a template in PCR amplifications. The conserved core region of WcE392-DSR gene was amplified by temperature-gradient PCR screening with degenerate Deg primers (S1 Table) [20]. Parallel PCR reactions were performed at different annealing temperatures ranging from 36.0 to 48.5°C (36.0; 36.1; 36.8; 37.8; 39.2; 40.7; 42.4; 44.0; 45.6; 46.9; 47.9 and 48.0°C) in the presence of 3 mM MgCl_2_. The conditions for the temperature-gradient PCR were: denaturation at 95°C for 5 min; 35 amplification cycles of 30 sec at 95°C, 45 sec at the annealing temperature and 1 min at 72°C; followed by a 7 min final extension at 72°C. Dynazyme II DNA polymerase (Thermo Scientific, Vantaa, Finland) was used for amplicons less than 1000 bp long. Phusion High-Fidelity DNA Polymerase (Thermo Scientific) was used for longer amplicons.

Sequences flanking the core region were obtained by inverse PCR essentially as described by Kralj et al. [21] (S1 Fig.). Briefly, genomic DNA was fragmented with several restriction endonucleases and subsequently self-ligated to create templates for PCR amplification with primers pointing outwards from the sequenced genomic region. *Kpn*I digestion and subsequent ligation produced a suitable template for the amplification of a 2555 bp fragment. Digestion with *Eco*RI, self-ligation and PCR yielded fragments extending to the flanking regions of the gene. The complete coding sequence of the WcE392-DSR gene was amplified with the primers 392dsrNcoF and 392dsrXbaR, thereby introducing an *Nco*I site with the start codon and 5´-sequence encoding a C-terminal 6× His-tag, followed by an *Xba*I site. The PCR product was cloned as an *Nco*I-*Xba*I fragment into pNZ8037 to yield the expression vector pDsrX, which was introduced to *L. lactis* NZ9800 by electroporation as described previously [22]. The pDsrX transformants were screened for by colony PCR with primers nisF and 392dsr3R [23]. Heterologous production of WcE392-rDSR and dextran was confirmed by streaking *L. lactis* (pDsrX) on a gM17 plate containing 10 µg/mL chloramphenicol, 4 ng/mL nisin and 20 g/L sucrose. The plasmid pDsrX was purified and the correct insert was verified by sequencing.

### WcE392-rDSR production and purification

For WcE392-rDSR production, 2 L of gM17 was inoculated with 10 mL of an overnight culture of *L. lactis* (pDsrX) and the culture was grown at 25°C with slow magnetic stirring. Expression was induced by adding 4 ng/mL nisin at an OD_600_ of ~0.3 and the culture was further incubated for 16 h at 25°C. The cells were separated by centrifugation for 20 min at 4000 g and 4°C. The supernatant was concentrated by ultrafiltration first to ~400 mL (6 ft^2^, Prep/Scale TFF, cut-off 10 kDa; Millipore, Bedford, MA) and then to 80 mL (Amicon 8400, cut-off 100 kDa; Millipore, Witten, Germany). The concentrated supernatant was supplemented with 300 mM NaCl, 2 mM imidazole and 50 mM NaH_2_PO_4_, pH 8.0, and loaded onto a 1 ml Ni-NTA Superflow column (Qiagen, Hilden, Germany). The column was washed and eluted following the manufacturers’ instructions with the exception that the imidazole concentrations in wash and elution buffers were 10 mM and 100 mM, respectively. The buffer of the enzyme sample was changed to 20 mM Na-acetate, pH 5.4, by ultrafiltration and dilution (Vivaspin 20, cut-off 10 kDa; Sartorius, Göttingen, Germany).

To synthesize dextran for characterizing and studying the enzymatic production of dextran in a wheat bran matrix, 5 L of culture supernatant containing WcE392-rDSR was partially purified by ultrafiltration as described earlier and diluted 1/200 with 20 mM Na-acetate, pH 5.4. The enzymatic activity of this preparation was determined by the Nelson-Somogyi assay as described in the subsection “Enzyme characterization”. SDS-PAGE analysis was carried out using a Criterion TGX 4–20% Stain Free precast Tris-HCl gradient gels (Bio-Rad, Hercules, CA) and Precision Plus Protein Unstained Standard.

### WcE392-rDSR sequence analysis

SignalP 4.1 (http://www.cbs.dtu.dk/services/SignalP/) algorithm was used for the identification of signal sequences in protein primary structure [24]. ProtParam tool on ExPASy server (http://web.expasy.org/protparam/) was used for computing molecular weight. Predicted protein structure for Wc392-rDSR was acquired with Phyre2 server (http://www.sbg.bio.ic.ac.uk/phyre2/) [25]. The computational structure was assessed using DaliLite (http://www.ebi.ac.uk/Tools/structure/dalilite/) [26]. Conserved domains were searched using the InterProScan tool (http://www.ebi.ac.uk/Tools/pfa/iprscan/).

### Enzyme characterization

The protein concentration of WcE392-rDSR preparation was determined by the DC Protein Assay Kit (Bio-Rad, Hercules, CA) with bovine serum albumin as the standard. The activity of WcE392-rDSR was assayed by ^14^C-sucrose radioisotope method, which is based on the incorporation of ^14^C-labeled glucose, derived from ^14^C-U-labeled sucrose, to the growing dextran chain [27]. The reaction mixture comprised 20 mM Na-acetate buffer, pH 5.4; 2 mM CaCl_2_; 146 mM ^14^C-U-sucrose (final radioactivity 41.7 kBq/mL, Perkin Elmer, Boston, MA) and 140 µg/mL of the enzyme. The reaction mixture was incubated at 35°C for 15 min, after which an aliquot was absorbed onto a 3MM filter paper (2.3 cm, Whatman). The filter was washed three times with methanol, dried and placed into Filter-count cocktail (Perkin Elmer, Groningen, the Netherlands). The radioactivity was determined using a Wallac 1414 liquid scintillation spectrometer (Wallac Oy, Turku, Finland). One activity unit was defined as the amount of enzyme that catalyzes the incorporation of 1 µmol of D-glucose into dextran in 1 min.

Alternatively, the WcE392-rDSR activity was assayed using the Nelson-Somogyi procedure [28, 29], which is based on the determination of the increase in the reducing value of the reaction mixture. The reaction mixture was the same as in the radioisotope assay with the exception of having unlabelled sucrose and a protein concentration of 3.5 µg/mL. D-fructose was used as the standard. One unit of activity was defined as the amount of enzyme that catalyzes the formation of 1 µmol of reducing sugar (fructose) in 1 min.

The Nelson-Somogyi method was used to determine the pH optimum, the effect of temperature on the activity, and the thermal stability of WcE392-rDSR. The buffers used for determining the pH optimum were 20 mM Na-acetate (pH 4.0 to 6.0) and 20 mM Tris-HCl (pH 6.5 to 7.0). All buffers were supplemented with 2 mM CaCl_2_. These reaction mixtures were incubated at 35°C for 15 min. The effect of temperature on WcE392-rDSR activity was determined at 25, 30, 35, 40 and 45°C for 15 min. Thermal stability was studied by incubating 350 μg/mL of the enzyme in 20 mM Na-acetate, pH 5.4, with 2 mM CaCl_2_, in the presence and absence of glycerol (0.5%, V/V) at 25, 35 and 40°C. Aliquots were withdrawn at 2, 6 and 24 h, and assayed for the remaining activity at 35°C. The results were plotted on a semi-logarithmic scale, log of the relative activity versus time, and the half-lives (*t*_1/2_) were calculated from the trend line slope, known as deactivation rate constant (*k*_d_).

Kinetic parameters were determined in 20 mM Na-acetate, pH 5.4, with 2 mM CaCl_2_, at 35°C by both the ^14^C-sucrose radioisotope and Nelson-Somogyi methods with the sucrose concentration ranging from 3 to 146 mM. The initial velocity data were analyzed by nonlinear regression using GraphPad Prism 6 (GraphPad, San Diego, CA).

After sucrose depletion, the leucrose (Santa Cruz Biotechnology, Santa Cruz, CA), glucose and fructose composition of the reaction mixture was analyzed by high performance anion exchange chromatography equipped with pulsed amperometric detector (HPAEC-PAD) as described by Pakarinen et al. [30].

### Dextran characterization

Dextran was synthesized *in vitro* by incubating 106 U (Nelson-Somogyi units) of the partially purified WcE392-rDSR in 100 mL of 20 mM Na-acetate, pH 5.4, containing 2 mM CaCl_2_ and 146 mM sucrose at 35°C for 24 h. Two volumes of ethanol were added and the solution was centrifuged at 10,000 g and 4°C for 15 min. The dextran pellet was re-dissolved in MilliQ water and precipitated and collected again before it was re-dissolved for freeze-drying. Dextrans produced by the native host *W. confusa* and the heterologous host *L. lactis* on MRS-S agar and gM17 agar with nisin, respectively, were extracted according to Maina et al. [7] and freeze-dried for parallel characterization.

For ^1^H nuclear magnetic resonance (NMR) spectroscopy analysis, dextran samples (10 mg/mL) were exchanged three times with D_2_O and transferred to NMR tubes (5 mm, Wilmad Ultra-Imperial, Aldrich Chemical Co.). The measurements were performed at 50°C using a 600 MHz Bruker Avance III NMR spectrometer (Bruker BioSpin, Rheinstetten, Germany). The chemical shifts were referenced to internal acetone (1H = 2.225 ppm).

Dextran solutions of 2.0 mg/mL were prepared in dimethylsulfoxide (DMSO) with 10 mM LiBr for the molar mass analysis. The solutions were kept at room temperature for at least four days for dissolution and then filtered using a 0.45 μm membrane filter before analysis. The high-performance size-exclusion chromatography (HPSEC) multidetector system (Maina et al. 2014) was used, with two Jordi xStream H_2_O Mixed Bed columns (250 × 10 mm, 5 μm, exclusion limit 10× 10^6^; Jordi Labs, Mansfield, MA) and a guard column of the same packing material (50 × 10 mm). The HPSEC data was processed with the Viscotek OmniSEC 4.5 software. A dη/dc value of 72 µL/g for dextran in DMSO-based eluent was used [31].

### Enzymatic dextran production in wheat bran

An experimental series was designed to optimize and model dextran production in wheat bran. The wheat bran with a particle size of 750 µm was autoclaved (120°C, 20 min) to inactivate its intrinsic enzymes. Water content (W), sucrose content (S) and incubation time (T) were selected as the variables and the amount of dextran produced (DX) as the response. A central composition design was used to arrange experiments and four replicates were made at the centre point to allow estimation of pure error at the sum of the square (Table 1). Upper and lower limits for variables were chosen on the basis of technological applicability. Water content was given as a percentage of total weight, whereas sucrose and dextran content were reported as percentages of dry weight (dw-%). Reactions were carried out in a total weight of 15 g at 30°C with an enzyme dosage of 37 U (Nelson-Somogyi units). The enzyme preparation was diluted into sterile tap water and the resulting liquid was mixed with the dry ingredients. The pH was measured from each reaction mixture at the end of the incubation. Reactions were stopped by freezing in liquid nitrogen. The samples before and after incubation were freeze-dried for sugar and dextran quantification. For glucose, fructose, sucrose and maltose analysis, the filtrate of water diluted sample from Amicon Ultra-0.5 centrifugal filter (Millipore Cork, Ireland) was determined by HPAEC-PAD according to Pakarinen et al. [32]. Dextran was analyzed according to the enzyme-assisted method described by Katina et al. [3].

**Table 1:** Enzymatic production of dextran into wheat bran.

**run**	**time (T, h)**	**water content (W,% w/w)**	**sucrose content (S, dw-%)**	**final sucrose content (dw-%)**	**final fructose content (dw-%)**	**final dextran content (DX, dw-%)**	**final pH**
1	2	70	10	0.9 ± 0.0	5.4 ± 0.2	5.4 ± 0.2	6.22
2	2	70	30	11.0 ± 0.8	8.6 ± 0.4	8.4 ± 0.3	6.15
3	2	90	10	-	5.8 ± 0.2	6.1 ± 0.2	6.35
4	2	90	30	-	14.8 ± 0.2	15.3 ± 0.5	6.33
5	6	70	10	-	6.2 ± 0.0	6.9 ± 0.0	6.27
6	6	70	30	-	13.9 ± 0.3	14.1 ± 0.2	6.25
7	6	90	10	-	6.0 ± 0.3	7.1 ± 0.0	6.25
8	6	90	30	0.9 ± 0.2	13.3 ± 0.5	14.8 ± 0.1	6.25
9	4	80	10	-	6.1 ± 0.3	7.1 ± 0.1	6.32
10	4	80	30	-	14.4 ± 1.0	14.9 ± 0.5	6.37
11	4	70	20	-	10.3 ± 0.3	11.5 ± 0.2	6.27
12	4	90	20	-	9.9 ± 0.1	11.0 ± 0.0	6.14
13	2	80	20	-	9.3 ± 0.1	8.5 ± 0.2	6.26
14	6	80	20	-	9.9 ± 0.6	9.0 ± 0.8	6.29
15	4	80	20	-	10.3 ± 0.5	11.4 ± 0.1	6.34
16	4	80	20	-	11.4 ± 0.7	11.8 ± 0.2	6.30
17	4	80	20	-	10.5 ± 0.3	11.8 ± 0.2	6.29
18	4	80	20	-	10.6 ± 0.4	11.5 ± 0.3	6.34

Composition of runs of the experimental design and the final dextran contents. Sucrose, fructose and dextran contents are given as a percentage of dry weight (dw-%). A hyphen (-) denotes that the values were below the detection limit.

The results were analysed by a partial least squares regression method. Dextran production was modelled by a quadratic model, which took into account the effects of variable alone, the effects of the interactions between two variables and the quadratic effects of variables alone. Regression analysis was calculated and the response surfaces were plotted with MODDE 9.0 software (Umetrics AB, Umeå, Sweden). The fit of model to the experimental data was given by the coefficient of determination, R^2^, which explains the extent of the variance that can be explained with the model. The predictive power, Q^2^, was calculated to measure of how well the responses will be predicted for a new experimental condition. Q^2^ is based on prediction of residual sum of squares. The pure error of the analysis, provided by the replicates at the experimental centre point, was used to predict model validity and lack of fit. Model validity is considered to be good, if it has a value of >0.25, which indicates no lack of fit (i.e. the model error is in the same range as the pure error). Reproducibility of the model was evaluated by comparing pure error to the total variation of the response.

### Baking of wheat bread supplemented with native and dextran-containing bran

Dextran was produced enzymatically into the wheat bran-water mixture (with water content of 73.5% and sucrose content of 16.7 dw-%). The experiment was carried out by mixing 293.8 g sterile tap water, 20 g partly purified WcE392-rDSR liquid (15 U/g, Nelson-Somogyi units), 17.7 g sucrose and 88.5 autoclaved wheat bran, and by incubating the mixture for 6 h at 30°C. Samples were taken at 0 and 6 h, and sugars, dextran content, pH and total titratable acids were analysed. For determination of titratable acidity (TTA), a 10 g sample was mixed with 100 mL of distilled water and the suspension was titrated to a final pH of 8.5 with 0.1 M NaOH (TitroLine Alpha 471217, Schott, Germany). TTA values are expressed as the volumes (mL) of 0.1 M NaOH consumed. TTA and pH analysis were done in duplicate.

Native and dextran-containing bran was used in wheat bread baking at 20% supplementation level. Native or modified bran was mixed with wheat flour, water, salt, sugar and fat for 4+6 minutes (Table 2 and Table 3). After a 10 min rest at 35°C and 75% relative humidity (RH) the dough was divided into 150 g pieces, rounded and moulded mechanically and proofed for 50 min (35°C/75% RH) in pans. The loaves were baked in a convection oven for 15 min at 200°C. After baking, the bread was cooled for 2 h before analysis. Bread with dextran-containing bran was compared to bread with native bran and to bread without bran. The compositions of the dough used for baking are presented in Table 2 and the processing protocol in Table 3. The major quality criteria for the different breads, specific volumes and instrumental hardness, were measured as described previously [3]. Means for the raw data were calculated and the significance of differences between samples was assessed using analysis of variance (ANOVA) with p ≤ 0.05. When the difference between the samples was significant according to ANOVA, pairwise comparisons between sample group means were made using Tukey’s test. All statistical analyses were performed with the R software.

**Table 2:** Compositions of the doughs used for studying the effects of bran with dextran on bread properties.

	**wheat bread**		**wheat bread with native bran**		**wheat bread with dextran-containing bran**	
	(g)	(%)	(g)	(%)	(g)	(%)
wheat flour	325	100	260	80	260	80
Water	208	64	240.5	74	26	8
Salt	5.85	1.8	5.85	1.8	5.85	1.8
margarine	9.75	3	9.75	3	9.75	3
dry yeast	4.88	1.5	4.88	1.5	4.58	1.4
milled wheat bran			65	20		
dextran-containing bran					298.35	90.6
total	553.5	170.3	585.98	176.3	578.53	184.8

**Table 3:** Dough processing parameters.

dough temperature	26°C
mixing (Diosna Spiral mixer)	4 min slow + 6 min fast
floor time	10 min (35°C and relative humidity 75%)
loaf weight	150 g
proofing in pans	50 min (35°C and relative humidity 75%)
Baking	15 min (200°C)

### Accession to WcE392-DSR sequence data

The WcE392-DSR gene sequence was deposited in GenBank with the accession number KJ173611.

## Results

### Identification, cloning and sequence analysis of WcE392-DSR

A PCR product of expected size (660 bp) was successfully amplified with DegF/R primers at an annealing temperature of 38°C. The fragment was sequenced and used as a starting point for inverse PCR (S1 Fig.). The full 4272 bp long Wc392-DSR gene was sequenced in this fashion. The WcE392-DSR open reading frame of 4272 bp corresponds to a polypeptide of 1432 amino acids. SignalP algorithm suggested the presence of signal peptide with a cleavage site between residues A42 and D43, which would result in a 1381 amino acid mature protein with a computational molecular weight of 152 kDa. The protein primary structure bears the closest resemblance to *W. confusa* LBAE C39–2 dextransucrase (Genbank: CCF30682.1, 93.3% identity) [15]. The search for conserved domains revealed cell wall binding repeats (PS51170) and overlapping glucan binding repeats (TIGR04035) located at the *N-* and *C*-terminals of the glycoside hydrolase family 70 (http://www.cazy.org/GH70.html) catalytic domain.

**Figure 1:**
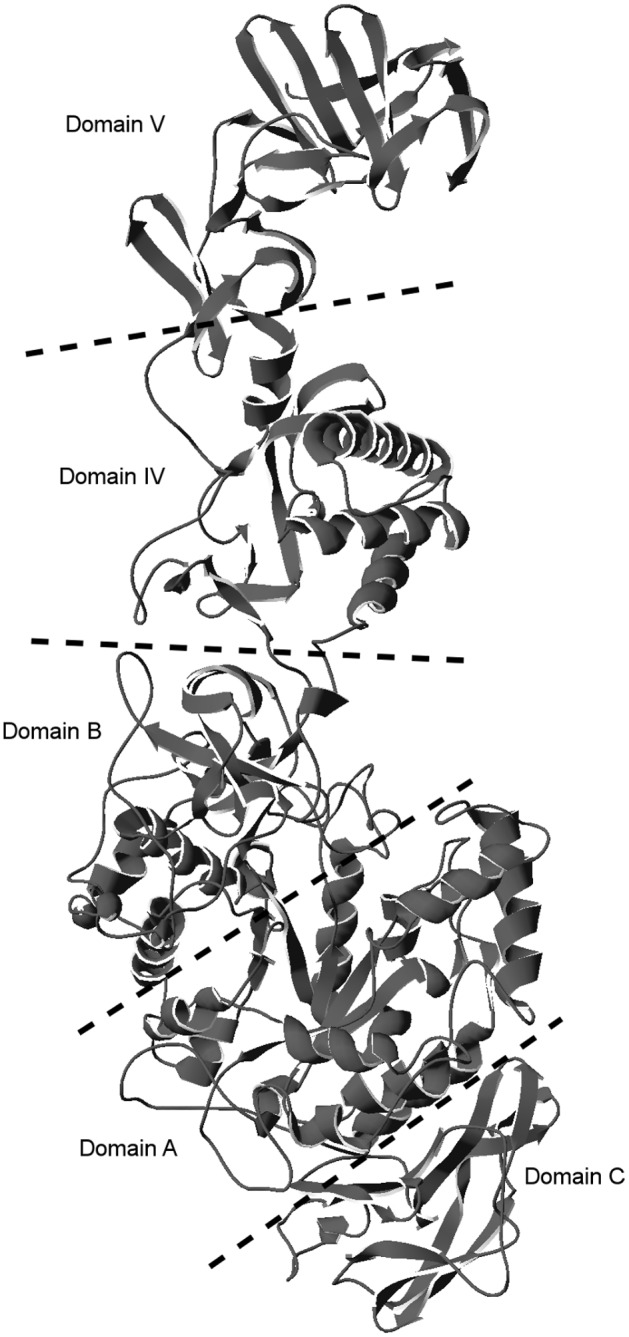
Predicted protein structure of WcE392-rDSR. Structural domains A, B, C, VI and V, as presented by Leemhuis et al.[1], are shown separated by dotted lines.

The tertiary structure for partial WcE392-rDSR (996 residues, K252 —R1247) was modelled using the Phyre2 server with the N-terminally truncated GTF180 from *Lactobacillus reuteri* 180 [33] as the template (Fig. 1). The modelled fragment has 51% amino acid identity to the template sequence and a 0.1 Å root-mean-square deviation of α-carbons between the computational model and the template. When the modelled part of WcE392-rDSR is compared to the *W. confusa* LBAE C39–2 dextransucrase, the differing residues are located mainly in the structural domain V (21/42 of the differing residues), to which glucan binding function is attributed[1]. Other substitutions are distributed between domains A (9 substitutions), B (1), C (5) and IV (6). Substitutions in the catalytic domain A are located on the protein surface and should not have an effect on the catalytic triad in the active site. A multiple sequence alignment of WcE392-dsr, *W. confusa* LBAE C39–2 dextransucrase and *Lactobacillus reuteri* 180 GTF180 is shown in S2 Fig.

### Expression of WcE392-rDSR gene and purification of the enzyme

The gene encoding WcE392-rDSR was cloned into the nisin-inducible expression vector. To detect WcE392-rDSR production, the *L. lactis* transformants were grown on gM17 agar supplemented with sucrose and nisin. Slimy colonies, resulting from dextran production, were formed within 16 h on this medium by the positive transformants. This suggested that the *L. lactis* expression system recognized the native *W*. *confusa* signal peptide and the enzyme was secreted into the growth medium.

**Figure 2:**
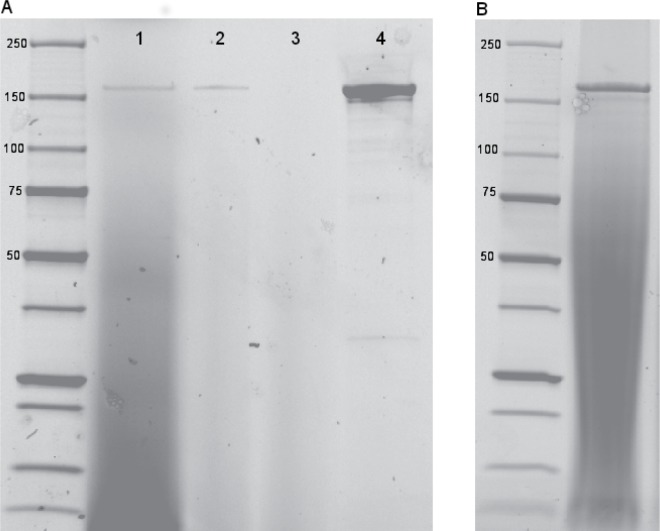
SDS-PAGE analysis of the purification of WcE392-rDSR. Gel A shows the purification protocol of the enzyme from *L. lactis* culture supernatant (1). A 2-fold concentrate obtained by ultrafiltration and the ultrafiltration permeate are shown on lanes 2 and 3, respectively. Lane 4 shows the sample eluted from Ni-NTA column. Gel B shows the approximately 60-fold concentrated enzyme sample obtained by ultrafiltration and dilution.

WcE392-rDSR was produced in a liquid culture and purified by nickel affinity chromatography to virtual homogeneity, as indicated by SDS-PAGE analysis (Fig. 2). A protein band was visible at ~160 kDa, which is in agreement with the molecular mass of 152 kDa calculated from the amino acid sequence. The supernatant containing WcE392-rDSR was also partially purified by ultrafiltration and dilution for enzymatic dextran production in bran matrix. In this sample, the WcE392-rDSR also presented a major band (Fig. 2).

### Enzyme characterization

The specific activity of the nickel affinity purified WcE392-rDSR was 7.2 ± 0.3 U/mg and 18.9 ± 0.6 U/mg, as determined by the ^14^C-sucrose radioisotope assay and Nelson-Somogyi assay, respectively. The Nelson-Somogyi method is based on measuring the increase in the reducing value of the reaction mixture resulting from fructose being liberated from sucrose in the transferase reaction synthesizing dextran. However, other carbohydrates present in the mixture also affect the measurement. These include glucose and fructose released in the hydrolytic reaction, the reaction by-product leucrose, and the polymerized dextran [34]. According to HPAEC-PAD analysis of the reaction mixture after sucrose depletion, glucose and leucrose accounted for only 2% and 5% (w/w) of total mono- and disaccharides. Fructose represented the remaining 93%, indicating that the transferase reaction comprises the main portion of the Wc392-rDSR activity.

**Figure 3:**
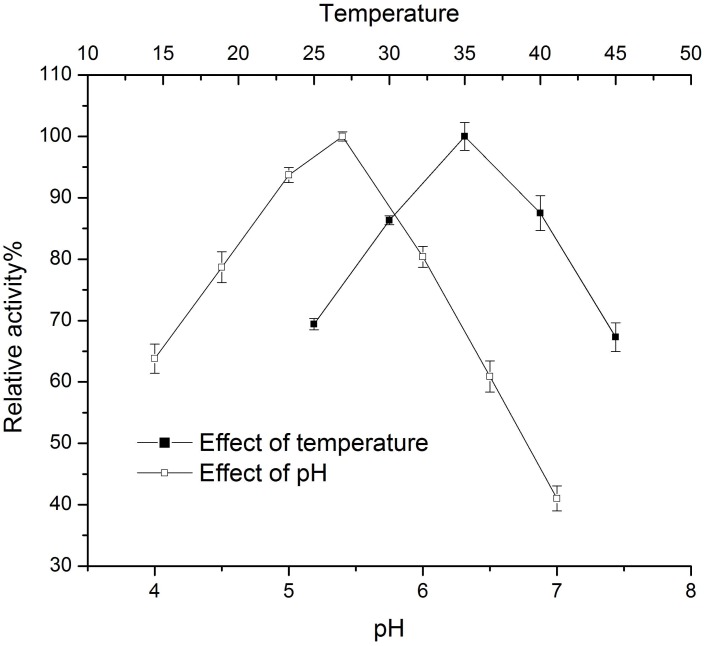
Effects of pH and temperature on WcE392-rDSR activity. Effect of pH was assayed in 20 mM Na-acetate (pH 4 to 6) and in 20 mM Tris-HCl (pH 6.5 to 7.0), and the effect of temperature in 20 mM Na-acetate, pH 5.4. Reactions were incubated for 15 min. The reaction mixtures were supplemented with 2 mM CaCl_2_ and the Nelson-Somogyi method was used for assaying the activities. Error bars represent the standard errors of the mean (n = 3).

**Figure 4:**
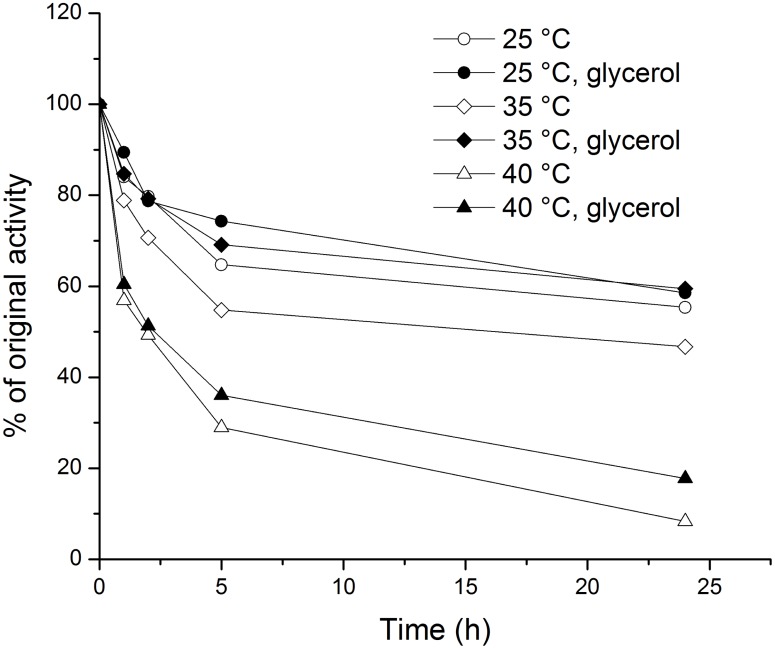
Thermal stability of WcE392-rDSR. The stability was measured in the presence and absence of 0.5% (v/v) glycerol, after incubation at 25°C, 35°C and 40°C for 1, 2, 5 and 24 h.

Since the transferase/hydrolase activity ratio of the dextransucrase can be expected to remain constant, the Nelson-Somogyi assay was considered adequate for determining the effects of pH and temperature on the activity. As shown in Fig. 3, the optimal pH for WcE392-rDSR activity was 5.4 in 20 mM Na-acetate buffer. The rate of dextran synthesis increased with higher temperatures up to 35°C, after which it decreased (15 min incubation). The thermal stability of WcE392-rDSR was studied at 25, 35 and 40°C in the presence or absence of glycerol up to 24 h (Table 4). The enzyme stability decreased sharply when the temperature was raised from 35°C (half-life of 26.3 h) to 40°C (7.4 h). Glycerol enhanced the stability at each of the three temperatures; for example, the enzyme stability at 35°C with glycerol was higher than at 25°C without glycerol (Fig. 4).

**Table 4:** Half-life (*t*_1/2_) of WcE392-rDSR in the presence and absence of glycerol (0.5%, v/v) at various temperatures in 20 mM Na-acetate buffer with 2 mM CaCl_2_, pH 5.4.

	**25°C**	**35°C**	**40°C**
no glycerol	33.0 h	26.3 h	7.4 h
with glycerol	41.3 h	37.5 h	11.2 h

WcE392-rDSR exhibited Michaelis-Menten kinetics with a *K*_m_ of 14.7 mM, a *V*_max_ of 8.2 µmol/(mg∙min) and a *k*_cat_ of 23.2 1/s, determined by using the sucrose radioisotope assay. The sucrose radioisotope assay and the Nelson-Somogyi assay gave similar *K*_m_ values, whereas values around 2.5 times higher were obtained for *V*_max_, *k*_cat_ and *k*_cat_/*K*_m_ with the Nelson-Somogyi method (Table 5).

**Table 5:** Specific activities and kinetic values of WcE392-rDSR.

**method**	**specific activity (U/mg)**	K_m_ **(mM)**	***V_max_*** **[µmol/(mg×min)]**	***k_cat_*** **(1/s)**	***k_cat_/K_m_*** **[1/(M×s)]**
^14^C-sucrose radioisotope assay	7.2 ± 0.3	14.7 ± 2.3	8.2 ± 0.4	23.2 ± 1.1	(1.6 ± 0.3) × 10^3^
Nelson-Somogyi assay	18.9 ± 0.6	13.0 ± 1.1	19.9 ± 0.6	56.4 ±1.7	(4.3 ± 0.5) × 10^3^

### Dextran characterization

Dextrans synthesized *in vitro* by WcE392-rDSR and *in vivo* by the native producer *W*. *confusa* VTT E-90392 and the heterologous host *L. lactis* NZ9800 (pDsrX) were isolated and characterized under the same conditions. The ^1^H NMR spectrum and corresponding structure of dextran from *W. confusa* VTT E-90392 has been reported previously [7]. The dextrans produced enzymatically and by the heterologous host *L. lactis* showed similar spectra (S3 Fig.) to that produced by *W. confusa* VTT E-90392. The anomeric proton signals were observed at 4.97 and 5.32 ppm for α-(1→6) and α-(1→3) linked residues, respectively. Based on their relative intensities, the ratio of α-(1→6) to α-(1→3) linkages was 97:3 in both cases and was equivalent to that reported for dextran from *W. confusa* VTT E-90392. The remaining proton resonances at 3.97, 3.91, 3.77, 3.73, 3.58, and 3.52 ppm were assigned to H-6b, H-5, H-6a, H-3, H-2, and H-4 of the α-(1→6)-linked residues, respectively. The signal at 4.19 ppm, representing H-5 of elongated branch point residue [-(1→6)-α-D-Glc*p-*(1→3)-], confirmed the presence of branches with more than one glucosyl unit [17]. DMSO with 10 mM LiBr was used as the solvent in the HPSEC analysis of dextran molar mass. DMSO was preferred because dextrans are prone to aggregation in aqueous solutions [35]. All dextrans showed similar molar masses in the range of 1.9 to 8.5 × 10^7^ g/mol.

### Optimization of enzymatic dextran production in wheat bran

**Figure 5:**
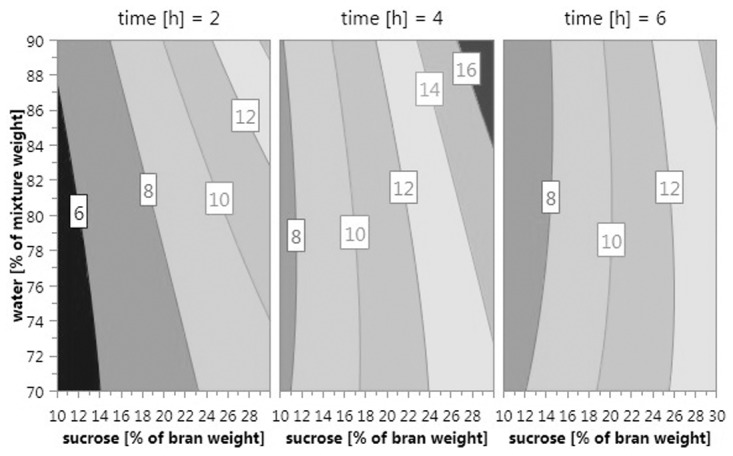
Influence of the sucrose and water contents in the initial reaction mixture and the incubation time on final dextran content in the experimental series shown as contour plots. Dextran production is enhanced by higher sugar content. In the 2 h plot, higher water content clearly increases dextran production but in the 4 h plot the effect can only be seen with higher sucrose content and in the 6 h plot the effect is completely muted. The quadratic effect of incubation time shows as highest dextran contents in the 4 h plot. Dextran amount is given as a percentage of dry weight.

HPAEC-PAD analysis showed that the wheat bran matrix contained up to 0.5 dw-% intrinsic glucose, fructose and sucrose, but no maltose. Eighteen experiments were performed according to the experimental design to study enzymatic dextran production in wheat bran by varying the content of sucrose, water and length of incubation. Sucrose was converted efficiently to dextran in most samples and no oligosaccharides were formed via the maltose acceptor reaction (Table 1). Dextran contents and pH values were in the ranges of 6.1–15.3 dw-% and 6.14–6.37, respectively (Table 1). Final fructose content was in agreement with the amount of dextran formed. Samples had a final glucose content of 0.2 dw-% or less. Based on the response values, a quadratic model was formed by excluding irrelevant terms to yield the following regression equation: DX = 0.0268S + 0.00614W + 0.00629T— 0.0107T^2^ + 0.00493W*S— 0.00493W∙T + 0.114. The model indicates that dextran production was most notably enhanced by higher sugar content (S). Water content (W) and incubation time (T) affected dextran production interactively. Incubation time was proposed to have a quadratic effect. Model-predicted dextran content as a response to model variables is depicted in Fig. 5. The model had a coefficient of determination R^2^ of 0.93 and coefficient of prediction Q^2^ of 0.62. Reproducibility of the model was 1, but the model validity indicated a significant lack of fit.

### Baking of wheat bread with dextran-containing bran

The technological effects of adding dextran-containing bran into wheat bread dough were studied using dough with and without native bran as the controls. Wheat bran supplemented with sucrose (16.7 dw-%) was incubated at 30°C for 6 h, during which all of the sucrose was consumed yielding a final dextran content of 8.1 ± 0.35 dw-%. Specific volume, hardness, pH and TTA values of the breads are presented in Table 6. The results indicate that the addition of native bran decreased bread volume and increased hardness leading to inferior quality of the wheat bread. The addition of dextran-containing bran into the dough resulted in a significant decrease in hardness both in relation to bread without bran (white wheat bread) and to bread with native bran. The hardness of bread with dextran-containing bran was 38% and 53% lower than that of bread without bran and bread with native bran, respectively. The volume of bread with dextran-containing bran was similar to wheat bread without bran and 10% higher in comparison to wheat bread supplemented with native bran. Wheat bread with native bran had slightly higher TTA than wheat bread, but the difference in pH was not significant. Bread supplemented with dextran-containing bran had slightly lower pH and higher TTA than the other breads.

**Table 6:** Bread textural properties, pH and total titratable acidity (TTA).

**bread**	**hardness (g)**	**volume (mL/g)**	**pH**	**TTA (mL^*a*^)**
wheat bread	244±18	3.78±0.03	6.01±0.06	2.70±0.02
wheat bread with native bran	320±15	3.41±0.07	6.18±0.02	3.25±0.02
wheat bread with dextran-containing bran	152±15	3.76±0.02	5.70±0.06	3.67±0.04

^a^Total titratable acidity values are given as mL of 0.1 NaOH.

## Discussion

Wheat bran is the most important by-product stream of wheat milling. Wheat bran components benefit health in several ways, including improving intestinal health, lowering blood pressure and reducing postprandial glycemic response. Even so, wheat bran is mainly used in low-value products such as livestock feed. In bakery products, applicability of wheat bran is limited because the bran particles interfere with gluten network formation leading to low specific bread volume and poor dough processability [36]. Dextran-producing *Weissella* strains have been demonstrated to have potential application in improving technological quality, including bread volume and dough processability, of various types of sourdough breads [3, 11]. In the present study, the *W. confusa* VTT E-90392 dextransucrase was cloned and characterized, and the potential of dextransucrase in preparing bran for use as a baking aid in high fibre baking was examined.

The conserved core sequence of WcE392-DSR gene was amplified with DegF/R primers under relatively non-stringent PCR conditions (low annealing temperature and elevated Mg^2+^ concentration). The complete coding sequence was acquired by inverse PCR. DegF/R primers have previously been used to screen for glucansucrase genes from *Weissella* species with inconsistent results—two studies report successful amplification [6, 37], while several efforts have been unsuccessful [5, 10, 38]. The degenerated forward primer DegF had two mismatching nucleotides when compared to the targeted site on *WcE392-DSR*, suggesting the DegF/R primers may be sensitive to competing annealing sites in the genome when screening *Weissella* species for glucansucrase genes. Previously, complete *Weissella* dextransucrase genes have been reported for four strains, *W. confusa* LBAE C39–2, *W. cibaria* CMU, *W. cibaria* LBAE K39 and *Weissella* sp. TN610 [6, 15, 16]. Based on sequence analysis, WcE392-DSR is very similar to *W. confusa* LBAE C39–2 DSRC39–2 and the amino acid substitutions observed are unlikely to cause drastic differences in catalytic properties. Most of the substitutions are located in domains attributed with glucan or cell wall binding functions which means the enzymes potentially differ in their adherence to cell surface or affinity for dextran.

*WcE392-DSR* was cloned into a nisin-inducible expression system in *L. lactis* that could excrete the recombinant protein into growth medium by recognizing the native *Weissella* signal peptide. With this system, protein aggregates, as experienced by Amari et al.[15], could be avoided and no additional fusion tags beside the 6 × His-tag were needed. The system also allows for dextransucrase and dextran production in a setting simulating microbial fermentation, which can be controlled by inducing gene expression with different levels of nisin.

Biochemical characterization of WcE392-rDSR showed that its optimal pH and temperature for activity assay were similar to other *Weissella* dextransucrases, which typically have a pH optimum of 5–5.4, and a temperature optimum of 30–35°C [6, 15, 16, 39, 40]. In a recently published article [41], native dextransucrase from *W. confusa* Cab3 shows stability up to 40°C and 0.5% glycerol was found to increase its half-life from 10.3 h to 74.1 h at 30°C. The quick inactivation above 40°C and the stabilizing effect of glycerol were also observed for WcE392-rDSR in this study.

The kinetics for two *Weissella* dextransucrases have been reported earlier. The recombinant rDSRC39–2 from *W. confusa* LBAE C39–2 and a native dextransucrase isolated from *W. cibaria* JAG8 follow Michaelis-Menten kinetics with *K*_m_ and *V*_max_ values of 8.6–13 mM and 20–27.5 µmol/(mg×min), respectively [15, 40]. However, the kinetics parameters of rDSRC39–2 dextransucrase were determined by 3,5- dinitrosalicylic acid (DNS) assay [15], which was pointed out to overestimate dextransucrase activity due to the significant over-oxidation of dextran and other carbohydrates in the sample, thus giving invalid enzyme assay and kinetics results [34, 42]. According to Vettori et al. [34], the DNS method generates a 5.8-fold higher activity than the ^14^C-sucrose radioisotope method. However, it is not known whether or to what extent over-oxidation exists in the Nelson-Somogyi reducing value assay. In this study, the ^14^C-sucrose radioisotope assay and the Nelson-Somogyi assay were compared for dextransucrase activity and kinetics measurements for the first time. The Nelson-Somogyi reducing value method gave about 2.5-fold higher specific activity and *V*_max_ than the ^14^C-sucrose radioisotope method, while the *K*_m_ values were similar regardless of the method used. As little free glucose and leucrose were formed as side products, the higher values produced by the Nelson-Somogyi method seem to be due to reactions with dextran. The kinetics for WcE392-rDSR (*K*_m_ of 13.0 mM and *V*_max_ of 19.9 µmol/mg/min) were comparable to those of the dextransucrase from *W. cibaria* JAG8 (*K*_m_ of 13 mM and *V*_max_ of 27.5 µmol/mg/min), which were obtained by the same Nelson-Somogyi method [40]. The *K*_m_ value obtained by the ^14^C-sucrose radioisotope method (18.9 mM) is similar to that of the glucansucrase from *Leuconostoc mesenteroides* NRRL B-1118 (12 mM), using the same method [27]. As the *V*_max_ value was not reported in that study, this study is the first to report a *V*_max_ of a glucansucrase obtained by the ^14^C-sucrose radioisotope assay.

The dextrans produced by WcE392-rDSR (*in vitro*) and by the heterologous host *L. lactis* exhibited high similarity to that produced by the native *W*. *confusa* as characterized by ^1^H NMR spectroscopy and HPSEC. All three exhibited similar structures and typical linkage composition for *Weissella* dextrans, which are less branched than dextrans from *Leuconostoc* strains such as *L. mesenteroides* [5, 7]. The molar masses reported for *Weissella* dextrans have varied between 10^6^ and 10^7^ g/mol [15, 35, 41]. Higher molar masses (10^7^–10^8^ g/mol) were obtained by HPSEC in this study. Significantly high molar mass (10^8^ g/mol) was also reported for dextrans synthesized *in vitro* with *Lc. mesenteroides* NRRL B-512F dextransucrase mutants applying asymmetrical flow field flow fractionation analysis [43].

The experimental design set up to optimize enzymatic dextran production in wheat bran showed that high sucrose content enhance dextran production, as expected. Higher water content is also expected to promote enzyme activity and this can be observed in the model with some incubation time values (Fig. 5).The model validity was poor, but the lack of fit is likely to be artificial as R^2^, Q^2^ and reproducibility of the model were good. The responses for the experimental center points used to determine the pure error were very close to each other and are not likely representative of the true experimental error, which caused the model error to be significantly larger than the pure error (S4 Fig.). The model proposed the optimal incubation time for maximum dextran content to be 4 h. As sugar results in Table 1 indicate that sucrose was completely converted in 4 h, longer incubation time did not result in higher content of dextran. A slight decrease in dextran content after 6 h may be due to either somewhat non-consistent dextran production in various experimental time points or slight degradation of dextran for unknown reasons. A reverse dextransucrase reaction, sucrose synthesis from dextran and fructose, is unlikely, as it has not been shown to our knowledge. The complete conversion in 4 h and the slight decrease in dextran content also contribute to the negative relationship between dextran production and the interactive factor of water content and time (W*T), which can be seen in Fig. 5. With shorter incubation time, higher water content clearly leads to higher dextran content but the effect is muted as incubation time is increased. In some of the samples, the final dextran concentration was exceeding the theoretical maximum. Dextran amount was measured with a method by Katina et al. [3] that was originally used for dextran quantification from wheat flour sourdough. The method includes a correction factor to account for dextran recovery from the sample matrix. The results of this study suggest that dextran is extracted more efficiently from bran than wheat sourdough and a wheat bran specific correction factor would have to be determined for more accurate results. Higher initial sucrose levels could have been tested to assess substrate saturation but the extreme values here were chosen based on technological applicability as high initial sucrose content means high final fructose content if the conversion is complete. High amounts of free fructose can lead to excessive browning and texture changes through the Maillard reaction in baking applications. Even though optimal conditions were not found in the experimental setting, the potential of efficient enzymatic dextran production in wheat bran was demonstrated even in low-water conditions.

Dextran-containing bran was further applied in a proof-of-concept wheat baking experiment. Dextran was produced enzymatically into wheat bran with reaction parameters falling into the range used in the modeling series. Addition of bran diminished bread volume and increased crumb hardness when compared to wheat bread without bran, confirming the negative impact of bran on bread quality observed by Katina et al. [44]. Production of dextran into bran compensated for the volume decrease seen in bread made with bran alone. Additionally, bread hardness was significantly decreased when using dextran-containing bran. The bread prepared with dextran-containing bran had a slightly lower pH and higher TTA than bread with no bran or with native bran. The slight acidification was possibly due to fructose fermentation by the baker’s yeast during dough proofing. Even though organic acids may have a negative impact on bread texture [9], the observation that the use of dextran-containing bran decreased crumb hardness in comparison to bread without and with native bran indicates that the slightly higher acid content did not have an unacceptable impact here. The slight acidity is neither expected to affect the flavor negatively since consumer acceptability of wheat breads is known to decrease only at pH < 4.9 [45]. In our previous study, dextran-containing wheat sourdough with pH 4.9–5.1 could be used at a high addition level (43% of the flour weight) in wheat baking without negative impacts on flavour or texture [3].

This is the first study to demonstrate the efficacy of *in situ* produced dextran without LAB fermentation in improving quality of high fibre wheat bread. Enzymatic production of dextran takes place in hours, compared to the 24 h of LAB fermentation typically needed for achieving high EPS concentrations [3, 9, 11, 46]. Furthermore, the use of enzyme instead of LAB fermentation does not lead to strong acidification, which may have a negative impact on product texture and flavour [9]. Lastly, it is important to note that as an enzymatic processing aid, the use of dextransucrase in bran processing doesn’t require end product labelling. These reasons collectively make dextransucrases an attractive alternative to LAB fermentation in bioprocessing of wheat bran for use in baking.

## Supporting Information

S1 Table PCR primers used for degenerate PCR, inverse PCR and cloning the Wc392-rDSR gene. Recognition sites of restriction endonucleases are underlined.
(DOCX)

S1 Fig Isolation of the WcE392-DSR complete coding sequence by inverse PCR. The initial fragment A was acquired by PCR with degenerate consensus primers targeting the conserved core region of LAB glucansucrase genes. Genomic DNA was then fragmented with several restriction endonucleases and self-ligated to circular fragments, from which the flanking regions could be amplified with primers pointing outwards from the known region (small black half arrows). Fragment B was obtained after treatment with *Kpn*I and fragments C and D were amplified after digestion with *Eco*RI. The complete coding sequence (large black arrow) together with a C-terminal His-Tag was cloned to expression host *Lactococcus lactis* NZ9800.
(EPS)

S2 Fig Amino acid sequence alignment of WcE392-dsr, *Weissella confusa* LBAE C39–2 dextransucrase DSRC39–2 and glucansucrase from *Lactobacillus reuteri* 180, truncated version of which was used as a template in structural modelling. The multiple sequence alignment is coloured by similarity. WcE392-dsr is compared to its closest homolog DSRC392 [15] and *Lactobacillus reuteri* 180 glucansucrase (UniProt ID: Q5SBN3). Q5SBN3 is shown in full length but the crystal structure was determined from an N-terminally truncated, fully functional form of the enzyme [33].
(TIF)

S3 Fig The 1D ^1^H NMR spectra of the dextrans produced by (A) the heterologous host *Lactococcus lactis* and by (B) WcE392-rDSR (*in vitro*). The spectra are similar to that of *Weissella confusa* E392 dextran produced *in vivo* [7]. The spectra were recorded at 600 MHz in D_2_O at 50°C. Peaks were referenced to internal acetone (1H = 2.225 ppm).
(TIF)

S4 Fig Regression of observed and predicted values of dextran content in the modelling experiment. Horizontal and vertical axes show predicted and observed dextran content, respectively, as proportion of dry weight. Data points are labelled by run number (Table 1).
(TIF)
