# Supplementary material for: Cloning and Characterization of a Weissella confusa Dextransucrase and Its Application in High Fibre Baking
Source: PLoS One. 2015 Jan 20;10(1):e0116418. doi: 10.1371/journal.pone.0116418 (PMC4300183; doi:10.1371/journal.pone.0116418)
Supplement: S1 Table — Recognition sites of restriction endonucleases are underlined. (DOCX) [file pone.0116418.s001.docx]

Table 1. PCR primers used for degenerate PCR, inverse PCR and cloning the Wc392-rDSR gene. Recognition sites of restriction endonucleases are underlined.

| primer | sequence | purpose |
| --- | --- | --- |
| DegF | GAYAAYWSAAYCCRYGTC | degenerate PCR |
| DegR | ADRTCCCRTARTAAVYKG | degenerate PCR |
| 392dsrNcoF | TTACCATGGCATGGGGATATGTTTTGGAA | *Wc392-rDSR* cloning |
| 392dsrXbaR | TAATCTAGATTAATGATGATGATGATGATGAATCGTCACTAGCTTACCAGACCCATTTGTC | *Wc392-rDSR* cloning, 6 × His addition |
| 392ds-1F | TGGTAAGATGGACCGCTTCTT | inverse PCR |
| 392ds-1R | ATTCGTCGCCATACCGTAAGC | inverse PCR |
| 392ds-2F | TCAGCTTCTTGGCGATGTGG | inverse PCR |
| 392dsRF-1F | TGGTGTGATGGCCACAGGTGA | inverse PCR |
| 392dsRF-1R | AGCACCACGGCCTTGGATTG | inverse PCR |
|  |  |  |
