# Supplementary figures and images for: Cloning and Characterization of a Weissella confusa Dextransucrase and Its Application in High Fibre Baking
Source: PLoS One. 2015 Jan 20;10(1):e0116418. doi: 10.1371/journal.pone.0116418 (PMC4300183; doi:10.1371/journal.pone.0116418)

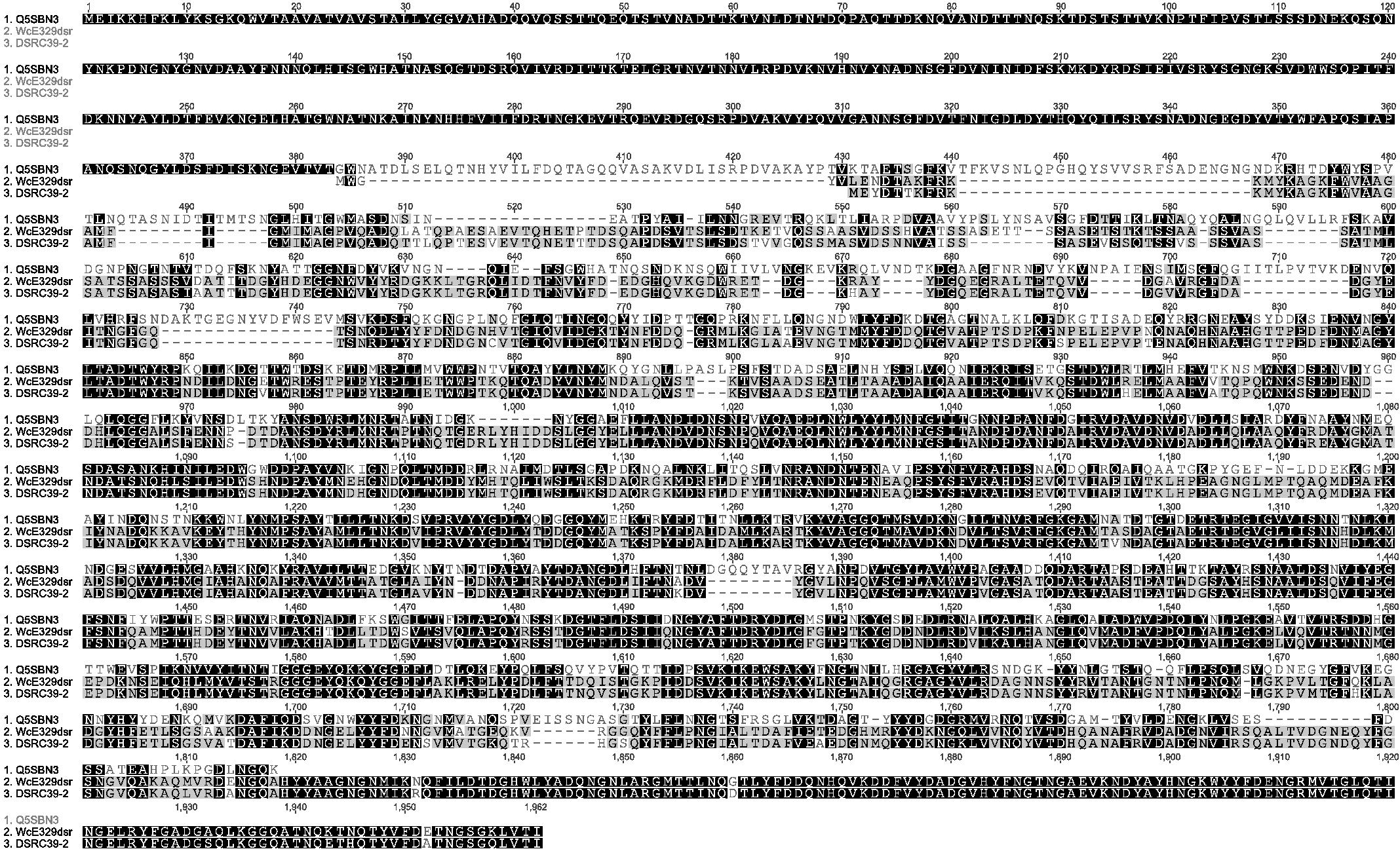

Supplement: S2 Fig — The multiple sequence alignment is coloured by similarity. WcE392-dsr is compared to its closest homolog DSRC392 [15] and Lactobacillus reuteri 180 glucansucrase (UniProt ID: Q5SBN3). Q5SBN3 is shown in full length but the crystal structure was determined from an N-terminally truncated, fully functional form of the enzyme [33]. (TIF) [file pone.0116418.s003.tif]

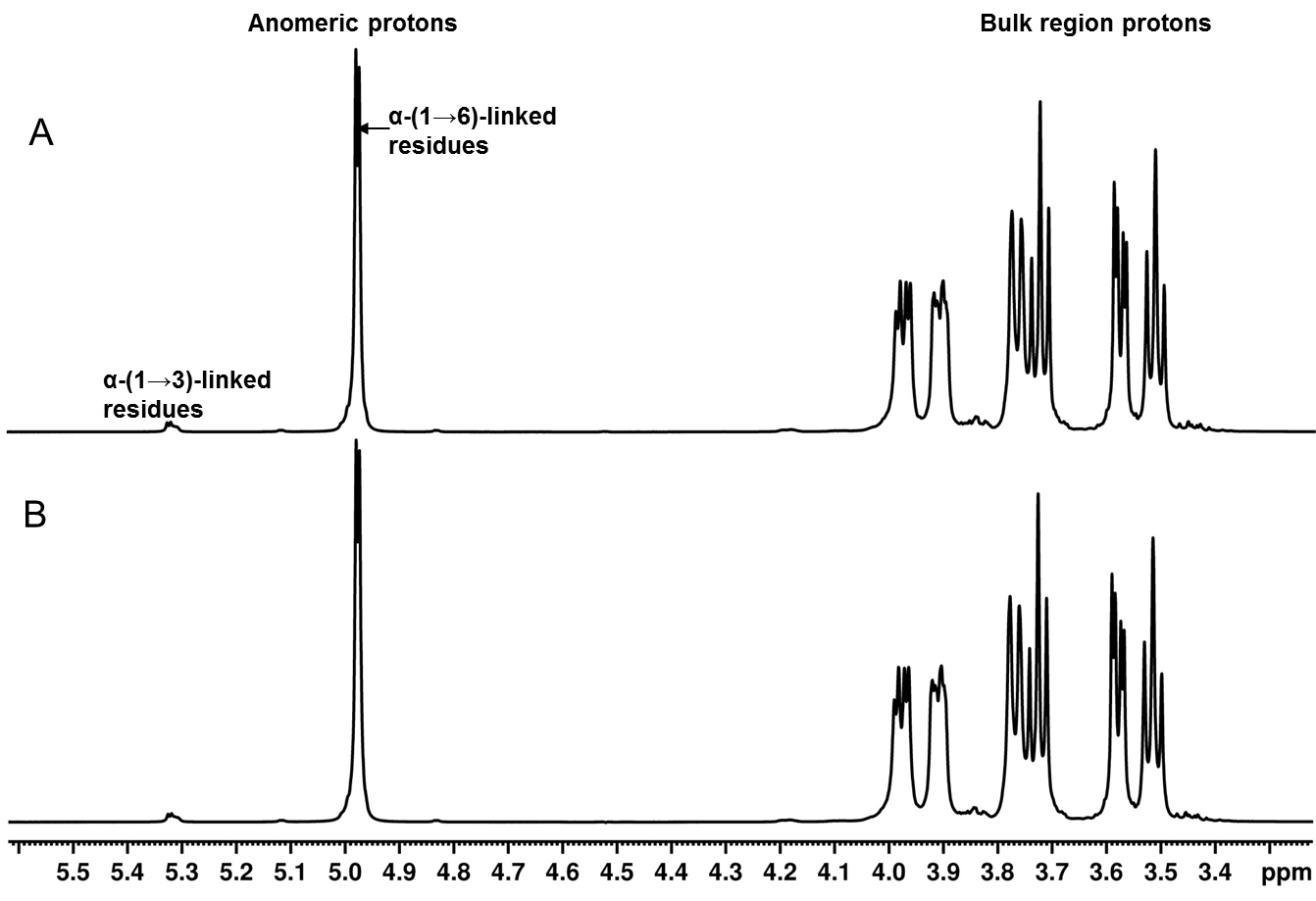

Supplement: S3 Fig — The spectra are similar to that of Weissella confusa E392 dextran produced in vivo [7]. The spectra were recorded at 600 MHz in D2O at 50°C. Peaks were referenced to internal acetone (1H = 2.225 ppm). (TIF) [file pone.0116418.s004.tif]

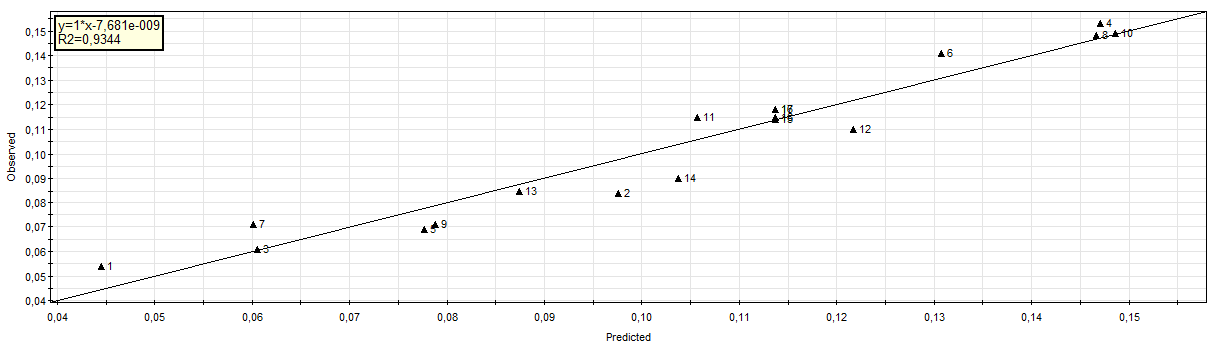

Supplement: S4 Fig — Horizontal and vertical axes show predicted and observed dextran content, respectively, as proportion of dry weight. Data points are labelled by run number (Table 1). (TIF) [file pone.0116418.s005.tif]
